# Effects of Noise Exposure and Aging on Behavioral Tone Detection in Quiet and Noise by Mice

**DOI:** 10.1523/ENEURO.0391-21.2022

**Published:** 2022-06-09

**Authors:** Kali Burke, Laurel A. Screven, Anastasiya Kobrina, Payton E. Charlton, Katrina Schrode, Dillan F. Villavisanis, Micheal L. Dent, Amanda M. Lauer

**Affiliations:** 1 Department of Psychology, University at Buffalo, The State University of New York, Buffalo, NY 14260; 2 Department of Otolaryngology–Head and Neck Surgery, Johns Hopkins University School of Medicine, Baltimore, MD 21205; 3 Department of Neuroscience, Johns Hopkins University School of Medicine, Baltimore, MD 21205

**Keywords:** age-related hearing loss, cochlear pathology, hearing in noise, noise-induced hearing loss, operant conditioning, threshold instability

## Abstract

Aging leads to degeneration of the peripheral and central auditory systems, hearing loss, and difficulty understanding sounds in noise. Aging is also associated with changes in susceptibility to or recovery from damaging noise exposures, although the effects of the interaction between acute noise exposure and age on the perception of sounds are not well studied. We tested these effects in the CBA/CaJ mouse model of age-related hearing loss using operant conditioning procedures before and after noise exposure and longitudinally measured changes in their sensitivity for detecting tones in quiet or noise backgrounds. Cochleae from a subset of the behaviorally tested mice were immunolabeled to examine organ of Corti damage relative to what is expected based on aging alone. Mice tested in both quiet and noise background conditions experienced worse behavioral sensitivity immediately after noise exposure, but mice exposed at older ages generally showed greater threshold shifts and reduced recovery over time. Surprisingly, day-to-day stability in thresholds was markedly higher for mice detecting signals in the presence of a noise masker compared with detection in quiet conditions. Cochlear analysis revealed decreases in the total number of outer hair cells (OHCs) and the number of ribbons per inner cell in high-frequency regions in aged, noise-exposed mice relative to aging alone. Our findings build on previous work showing interactions between age and noise exposure and add that background noise can increase the stability of behavioral hearing sensitivity after noise damage.

## Visual Abstract

**Figure:**
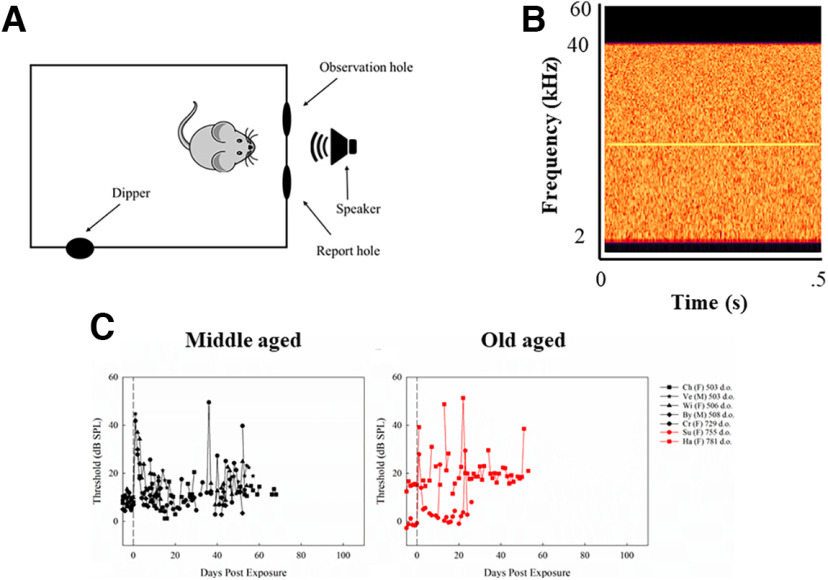

## Significance Statement

Hearing in noisy environments is diminished after exposure to noise and with age. Age also increases the susceptibility of the auditory system to dysfunction and may increase susceptibility to or recovery from damaging noise exposures. We investigated behavioral detection of pure tones in quiet and noise by aged, noise-exposed mice. Our results demonstrate increased susceptibility to temporary threshold shifts and diminished long-term recovery after noise exposure with increased age. Surprisingly, we observed increased performance instability when listening in quiet compared with noisy backgrounds in aged, noise-exposed mice. Presumably, this result reflects the activation of top-down mechanisms (e.g., the auditory efferent system) that enhance hearing in noise.

## Introduction

Age-related hearing loss is a major public health issue that is thought to be due to the interaction between natural aging processes, genetic susceptibility, diseases, and the accumulation of ototoxic and environmental exposures, although the effects of interactions between these various factors on age-related perceptual deficits have not been well characterized (Salvi et al., 2001). A history of noise exposure is associated with more severe cochlear pathology and poorer hearing outcomes in humans (PZ Wu et al., 2020, 2021; Gopinath et al., 2021). However, studies in humans report difficulty determining the overall effects of noise exposure with age because of several confounding factors related to the environment and differences in genetic susceptibility (Toppila et al., 2009; Sliwinska-Kowalska et al., 2006).

Susceptibility to damage from loud noise also depends on age in mouse models (Henry, 1982; Li, 1992; Li and Borg, 1993; Ohlemiller et al., 2000; Ho et al., 2014) and is increased in strains with genetic mutations associated with early-onset age-related hearing loss (Erway et al., 1996; Holme and Steel, 2004). Exposure to noise early in adulthood results in temporary threshold shifts that accelerate the progression of damage to the auditory periphery with age (Kujawa and Liberman, 2006; Fernandez et al., 2020). Yet, perception of tones in noise is not necessarily impaired by conditions that selectively damage the auditory nerve (Henry and Abrams, 2021; Resnik and Polley, 2021; Henry, 2022). Young-adult brains are adept at increasing central gain to restore sensitivity to sounds after noise-induced peripheral damage (Salvi et al., 2003; Gold and Bajo, 2014; Chambers et al., 2016; Clarkson et al., 2016; Zhao et al., 2016; Schrode et al., 2018), whereas compensatory central gain is impaired in aged animals exposed to noise, resulting in diminished subcortical responses to sound onsets (McFadden and Willott, 1994; Möhrle et al., 2016; Knipper et al., 2020).

Evoked potential measurements are typically used to quantify hearing loss in animal models; however, hearing loss is more typically assessed with behavioral measurements in humans. Hearing measured behaviorally is typically 5–30 dB more sensitive than what is measured physiologically (Dent et al., 2018; Burke et al., 2021), which may be due to anesthesia, strain, age, sex, and stimulus characteristics. Recent experiments have shown that changes in auditory brainstem response thresholds and waveform morphology precede behavioral hearing loss onset but correspond to substantial outer hair cell (OHC) and auditory nerve synapse degeneration in the CBA/CaJ mouse model (Henry, 2004; Kobrina et al., 2020). Furthermore, the long-term stability of hearing loss is rarely assessed behaviorally in animal models, but models of early-onset and late-onset age-related hearing loss suggest differential effects on the stability of hearing in quiet and noise over time (Prosen et al., 2003; Kobrina et al., 2020). The long-term effects of aging and noise interactions on auditory function have not been thoroughly studied in mammalian models (for review, see Lauer et al., 2019).

To investigate long-term behavioral recovery and stability after noise exposure in aging CBA/CaJ mice, we tracked behavioral detection of pure tones in quiet and masked environments after acute noise exposure in middle-aged and old-aged adult mice. We chose exposure conditions that were previously used to induce cochlear synaptopathy in CBA/CaJ mice with the intention of inducing increased synaptopathy beyond what occurs with natural aging and because this exposure is thought to primarily affect hearing in noise (Kujawa and Liberman, 2015). We hypothesized that the age of the mouse would affect the susceptibility to noise-induced hearing deficits, with middle-aged mice having fewer deficits than old-aged mice. We also hypothesized that hearing deficits would be worse in noise than in quiet after noise exposure. Cochlear specimens were examined for patterns of damage following the behavioral experiments. We show that detection of tones in quiet and noise is generally worse immediately after noise exposure, but middle-aged mice tend to recover over time more than old-aged mice. We also show that day-to-day variability is lower for tone detection in noise compared with quiet. Cochlear synaptopathy was greater than expected based on aging alone, but only in the high-frequency cochlear regions. Hair cell loss also occurred in the OHCs in high-frequency regions.

## Materials and Methods

### Experimental animals

Twenty-eight adult CBA/CaJ laboratory mice (*Mus musculus*, IMSR catalog #JAX:000654, RRID: IMSR_JAX:000654, obtained from: https://www.jax.org/strain/000654) ages 414–844 d old (d.o. hereafter) at noise exposure were used in this experiment. Mice were matched by sex (13 male and 15 female) to avoid sex bias (Lauer and Schrode, 2017; Villavisanis et al., 2018, 2020). Most mice entered the experiment after participating in pure tone behavioral discrimination studies in our laboratory. Some young mice entered the experiment at postnatal day (P)60 and did not participate in any other experiments. Training of all mice began when mice were approximately two to three months old and the experiments lasted up to 1000 d.o. (see Results). The original breeding pairs were acquired from The Jackson Laboratory. Our mice were bred in-house at the University at Buffalo, the State University of New York (SUNY), and all procedures were approved by the University at Buffalo (SUNY) Institutional Animal Care and Use Committee. All animal experimentation was conducted in accordance with the Society for Neuroscience’s Policies on the Use of Animals and Humans in Neuroscience Research. All mice were singly housed and kept on a reverse 12/12 h light/dark cycle (with lights off at 6 A.M. and on at 6 P.M.). Mice were tested during the dark portion of this cycle for 1 h, at the same time of day, 6–7 d a week. Mice were water restricted and kept at ∼90% of their free feeding/drinking weights throughout the duration of the experiment. To maintain weight and performance, old mice (over 900 d.o.) were allowed free access to a water bottle. All mice had unrestricted access to food, except while participating in the experiment.

### Behavioral apparatus

Mice were tested in one of six identically constructed testing booths. The mice were placed into a wire cage (23 × 39 × 15.5 cm) within a sound-attenuated chamber (53.5 × 54.5 × 57 cm) lined with 4-cm-thick Sonex sound attenuating foam (Illbruck Inc.). The chamber was illuminated at all times by a small lamp (Hampton Bay, SKU 208826) with an 8-W light bulb, and the behavior of the mice during test sessions was monitored by an overhead web camera (Logitech QuickCam Pro, Model 4000). The test cage consisted of an electrostatic speaker [Tucker-Davis Technologies (TDT), Model ES1], a response dipper (Med Associates Model ENV-302 M-UP), and two nose poke holes surrounded by infrared sensors (Med Associates Model ENV-254). The experiments were controlled by Dell Optiplex 580 computers operating TDT modules and software. Stimuli were sent through an RP2 signal processor, a PA5 programmable attenuator, an ED1 electrostatic speaker driver, and finally to the speaker. Inputs to and outputs from the testing cages were controlled via RP2 and RX6 processors. Power supplies were used to drive the dippers (Elenco Precision, Model XP-650). Custom MATLAB and TDT RPvds software programs were used to control the hardware. For masked noise experiments, a dome tweeter was placed next to the ES1 speaker for noise presentation (Model FT28D, Fostex, Madisound). The continuous white noise was generated using a noise generator (Model 3025, ACO Pacific Inc.).

### Stimuli

The stimuli for this experiment were 14- and 20-kHz pure tones (500 ms in duration with 5-ms rise/fall cosine ramps). These pure tone stimuli were selected because they surround the peak sensitivity of hearing for this strain of mice (16 kHz; reported by Kobrina and Dent, 2016). The pure tone stimuli were created and edited in Adobe Audition (v.5; RRID:SCR_015796). Sound pressure levels for the 14-kHz pure tone were calibrated using a sound level meter (Larson Davis, System 824) with the microphone placed at the approximate location where the mouse’s head would be during testing. Calibrations were conducted weekly. Sound pressure levels for the 20-kHz pure tone were calibrated using an ultrasound recording system (Avisoft, Model USG 116-200) and a custom MATLAB script, again with the microphone (Avisoft Bioacoustics, Ultra Sound Gate CM116) placed at the approximate location where the mouse’s head would be during testing, and were conducted weekly.

The white noise masker was generated using an ACO Pacific Extremely Random white noise generator. This generator produces white noise that recycles every 2 d, making the repetition of a cycle of noise far enough apart to avoid mice potentially memorizing the noise content. The frequency range of this masker falls between 1.6 Hz and 39 kHz, completely overlapping with both tone frequencies tested here. The 1/3 octave band level surrounding 14 kHz was 40.8 dB and surrounding 20 kHz was 34.4 dB. The masker was presented at an overall intensity of 60 dB SPL and was calibrated weekly with the Larson Davis (System 824) sound level meter.

### Behavioral procedure

Mice were trained using a Go/No-go operant conditioning procedure on a detection task. When the mouse initiated a trial by poking to the observation nose-poke hole, a variable waiting interval ranging from 1 to 4 s began. Following the waiting interval, a single test stimulus was presented. Using signal detection theory, a hit was recorded if the mouse poked in the report nose-poke hole within 2 s of the onset of the target and received a reinforcement of 0.01 ml of milk chocolate Ensure. If the mouse failed to report the presence of the sound when one was presented, a miss was recorded and the trial ended. To confirm that mice were not randomly receiving hits by chance, sham trials were presented 30% of the time to monitor whether mice remained under stimulus control. In this case, the mouse initiated a trial at the observation hole, but no stimulus was presented. If the mouse continued to remain in the observation hole, this was marked by a correct rejection and the next trial would commence. If the mouse moved to the report hole when no sound was presented, this was marked as a false alarm, and it was punished with a short blackout period, during which mice were unable to initiate a trial (1–6 s). Sessions in which mice did not maintain both a mean hit rate above 80% across all targets and a false alarm rate below 20% were excluded from the data analysis.

Mice typically completed between 50 and 400 trials per testing session, and shorter sessions were included in the analysis so long as hit and false alarm rates were both at criteria. A single pure tone frequency, presented in quiet or in a white noise background was selected for each mouse, and they were only tested on this stimulus for the entirety of the experiment. Throughout each session, only stimulus level varied from trial to trial. Within a session, all of the stimuli were presented to the mice in randomized blocks of 10 (seven targets of varying intensities and three shams per block). All stimuli were presented to mice according to the Method of Constant Stimuli. Once performance stabilized for very loud sounds, two or three out of the seven targets were attenuated in steps of 5–10 dB depending on the age and performance of the mouse, such that mice consistently missed one of the quietest two targets over 50% of the time and the second or third target was over 50% correct. Four hundred trials were then collected and pre-exposure thresholds were calculated from the final 200 trials. Once a stable threshold was calculated for that tone frequency, mice were exposed to the acute noise described below. Data collection continued for at least 45 d, until mice recovered to baseline or stabilized, after which point the mice were anesthetized with a 200 mg/kg ketamine and 10 mg/kg xylazine mixture diluted in sterile water with a volume corresponding to their body weight (e.g., a 20-g mouse received 0.2 ml of anesthetic solution) and perfused via the heart with 60 ml of either 4% paraformaldehyde or a 2% glutaraldehyde 2% paraformaldehyde mixture. Cochleae were re-perfused via the oval and round windows with these fixative agents and tissue was sent on for further anatomic analyses to measure the number of inner hair cells (IHCs) and OHCs, as well as the number of ribbons per IHC and OHC.

### Noise exposure

After stable baseline pre-exposure thresholds were reached across 5 d as described above, mice were placed into a standard plastic mouse cage (30 × 19 × 13 cm) lined on the bottom with lab-grade shavings. All other enrichment was removed from the inside of the cage and the food was removed from the cage top. The mouse cage was then placed inside of a custom-built soundproof chamber (53.5 × 54.5 × 57 cm) lined with 4-cm-thick Sonex sound attenuating foam (Illbruck Inc.). A Fostex dome tweeter speaker (FT28D), mounted atop a chicken wire bridge (0.5 × 0.5-inch squares) was suspended over the center of the mouse cage in the booth. The Fostex speaker was wired to an Amplifier (Crown D-75A), which was hooked up to a Dell Optiplex computer (Model 580). The noise for the exposure was a narrowband white noise segment with a frequency band of 8–16 kHz, similar to what has been used in other studies (Kujawa and Liberman, 2015). The sound file was 5 min in duration and was presented on a continuous loop for the duration of the noise exposure (2 h). Mice were allowed to freely roam their cage throughout the exposure. Calibrations were conducted using a Larson Davis sound level meter (System 824) to ensure the noise was 100 dBA (fast scale) in all four corners and in the center of the cage. Calibrations took place before every noise exposure.

### Inner ear immunohistochemistry

Mouse cochleae were fixed for 1 h and decalcified for at least 14 d in 1% EDTA. The organ of Corti was dissected into five to six flat turns according to methods from the Eaton Peabody Laboratories. Cochlear pieces were blocked for 1 h in the following solution to inhibit nonspecific binding: 5% normal goat serum, 10% bovine serum albumin, 0.5% Triton X-100 (Electron Microscopy Services), in PBS using routine procedures previously used by our laboratory (Schrode et al., 2018; Kobrina et al., 2020). The blocking buffer was removed and replaced with diluted primary antibody mixture for 24 h at 4°C with mouse mono-clonal anti-CTBP2 to label ribbons (1:200, BD Biosciences, catalog #612044, RRID:AB_399431), rabbit polyclonal anti-myo6 to label hair cells (1:500, Sigma-Aldrich, catalog #M5187, RRID:AB_260563). Cochlear pieces were rinsed 24 h later and incubated in secondary antibodies in blocking buffer for 2 h at room temperature, followed by an additional rinse. The secondary antibodies were Alexa Fluor 568 goat anti-rabbit (1:1000, Thermo Fisher Scientific, catalog #A-11036, RRID:AB_10563566) and Alexa Fluor 488 goat anti-mouse (1:1000, Thermo Fisher Scientific catalog #A-10667, RRID:AB_2534057). The cochlear pieces were then mounted in Fluoromount-G (Southern Biotech) on subbed slides with coverslips.

The number of ribbons and hair cells were quantified at nine frequency regions (4, 5.6, 8, 11.2, 16, 22.6, 32, 45.2, and 64 kHz) along the cochlea in 100-μm sections. Locations were identified using low magnification ∼5× photographs and the ImageJ (RRID:SCR_003070) plugin Measure_Line, developed by the Eaton Peabody Laboratories. It should be noted that this reflects the cochlear place-frequency map in normal, young adult mice, and the noise exposure is likely to have shifted the place-frequency map (Müller et al., 2005). We collected z-stacks at 63× magnification using one of two confocal microscopes depending on availability (LSM 700 Axio Imager 2; Carl Zeiss or LSM 710 Zeiss Axio Observer, Carl Zeiss). Maximum intensity projections were created from confocal z-stacks at a step size of 0.25 μm to capture the entire thickness of each hair cell and its associated labeling. In Fiji (ImageJ) software (RRID: SCR_002285), we counted the number of hair cells and associated afferent ribbons within a 100-μm window corresponding to each frequency-specific point. Cells and their ribbons were only included if the entire cell was visible in the image frame. Total number of CTBP2-positive ribbons were counted and divided by the total number of hair cells within each frequency.

### Data analysis

#### Behavior

Data for the detection of tones in quiet and in the presence of a masker were analyzed separately for each tone frequency. Given the longitudinal design of this study, as well as the repeated sampling for each mouse and several missing data points, a linear mixed-effects model was used to analyze the results. Data were pooled across the sexes due to the small sample size which would not allow us to draw valid conclusions about sex differences and to improve statistical power.

Thresholds (dB SPL) were used as the dependent variable to examine each tone frequency (14-20 kHz) and masker status (masked or quiet) separately. Each model was compared across middle-aged and old-aged mice, with middle-aged mice ranging from 365 to 730 d.o. and old-aged mice ranging from 731 to 1095 d.o. These age groups were selected based on previous work showing that with advanced age (e.g., over ∼700 d.o. in mice), hearing starts to decline rapidly (Kobrina and Dent, 2020; Kobrina et al., 2020). We then used the model to compare thresholds across our age groups for each stimulus over time. Time was binned into time periods for the analyses in six windows to minimize the number of critical comparisons and to avoid an inflation of Type I error by comparing every single day. Any days before the noise exposure were classified as baseline, then the results were classified as postexposure periods of 1–5, 6–10, 11–20, 21–40, and >40 d. Whether thresholds in our age groups differed across the time bins was assessed using the following model: lmer(threshold (dB SPL) ∼ age group × time period+ (1|Id)) (*lmer* in the *lme4* R package, RRID: SCR_015654). We validated the use of our model by testing against an intercept-only model which uses mouse identity as a predictor. We then proceeded to test the goodness of fit of our model against other models using the step-up method of linear mixed-effects modeling. The goodness of fit for our model was measured using Akaike information criterion (AICc) with a criterion specifically used to evaluate goodness of fit for data with small sample sizes. The thresholds for quiet and masked tone detection were evaluated in separate models for 14 and 20 kHz. For the model predicting thresholds for 20 kHz in quiet, the factor of age group was excluded from the model as it did not improve the goodness of fit. *Post hoc* comparisons using Tukey’s method were performed to assess the relationships between age group and time period, with the *p* values adjusted to the number of family-based comparisons to reduce Type I error (corrected *p *=* *0.0014 for 14 kHz, quiet; 14 kHz, masked; and 20 kHz, masked; corrected *p *=* *0.003 for 20 kHz, quiet; *emmeans* R package, RRID: SCR_018734).

Additionally, daily thresholds for each mouse were used to calculate a mean deviation variability index for each mouse after noise exposure (which is preferable to reporting the standard deviation when data are not normally distributed). Using the interquartile range (IQR), outliers were identified as values outside the range Q1–1.5*IQR to Q3+1.5*IQR using boxplot.stats in base package R, which removed two mice. Comparisons before and after noise exposure were not conducted because of the extremely limited number of samples taken before noise exposure. In Kobrina et al. (2020), behavioral day-to-day detection performance stability in quiet and noise was assessed across the lifespan and revealed that performance stability generally varied between 0.1 and 10 dB across conditions, ages, and subjects, but that performance was generally more stable with age. Performance stability was not significantly different between quiet and noise background conditions. The Kobrina et al. (2020) study was performed in unexposed aging mice under similar conditions to this experiment and thus we did not expect differences across groups at baseline in performance stability. An ANOVA was conducted to examine how the day-to-day variability after noise exposure was affected by the interaction between masker status and stimulus. Linear regression analyses were also conducted to explore whether the age of the mouse could predict changes in day-to-day variability across conditions [similar to what is shown by Kobrina et al. (2020) without noise exposure].

#### Cochlear anatomy

To determine the effect of noise exposure on cochlear anatomy, we used linear mixed-effects models (*lmer* in the *lme4* R package, RRID: SCR_015654) to assess the effects of age and frequency on CTBP2-positive ribbon counts per IHC and OHC and number of IHCs and OHCs per 100 μm. Frequency, age, and exposure group were all treated as categorical factors. Subjects in the exposed (*N* = 5) and unexposed groups (*N* = 10: *N* = 3 current study, *N* = 7; Kobrina et al., 2020) were age-matched and thus age did not improve the model fitness and results were averaged across age. We controlled for repeated measures dependencies in our data by including a random intercept for mouse identity. We present the results for the ANOVA based on each model, as well as *post hoc* tests controlling for multiple comparisons using the mvt adjustment (*emmeans* R package, RRID: SCR_018734). Tukey’s *post hoc* analyses were performed to assess significance (*emmeans* R package, RRID: SCR_018734).

## Results

### Behavior, quiet

#### 14 kHz in quiet

**Figure 1.**
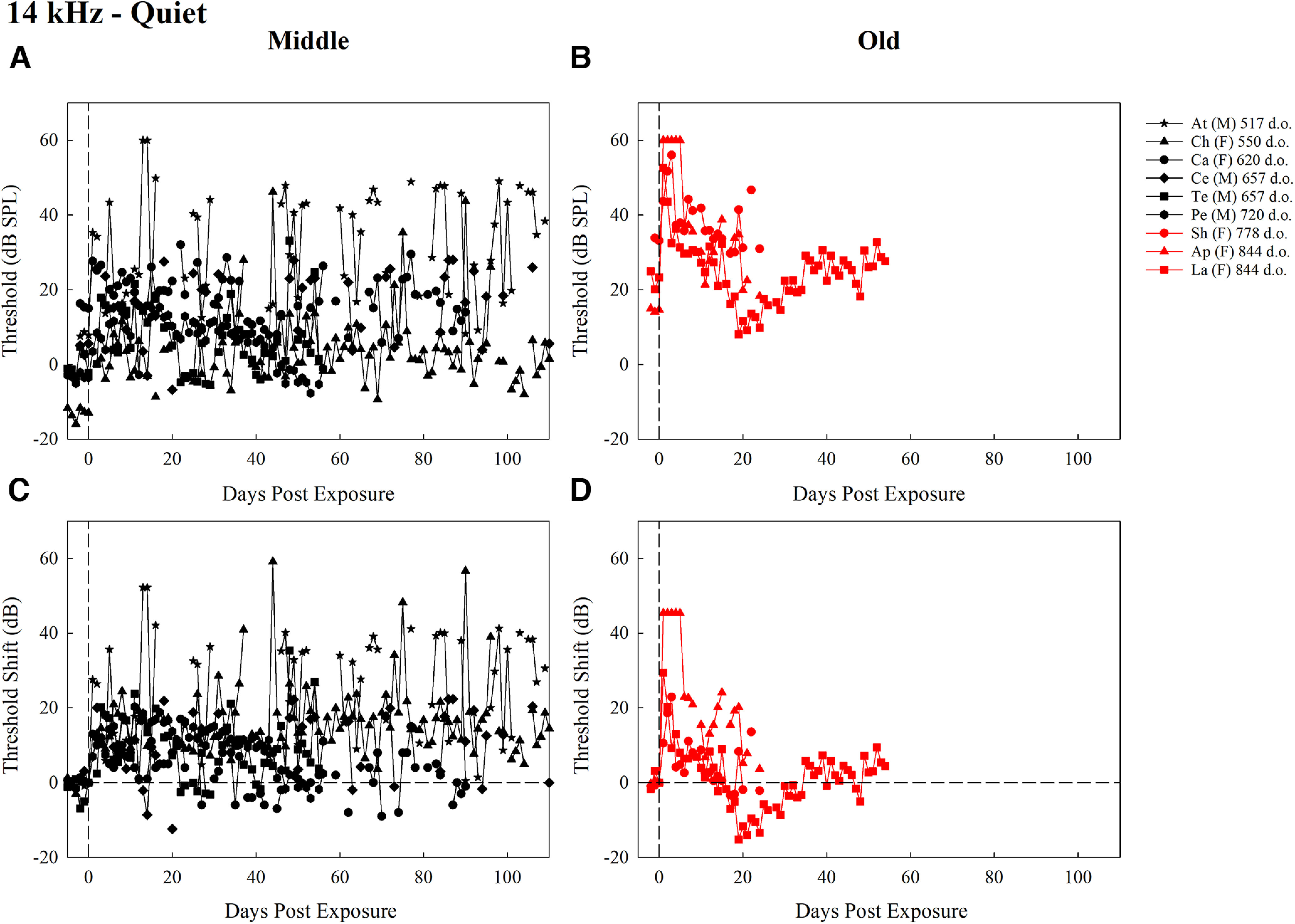
Thresholds (dB SPL) for middle-aged mice (***A***) and old-aged mice (***B***), and threshold shifts (dB) for middle-aged mice (***C***) and old-aged mice (***D***), trained to detect 14-kHz tones in quiet for all test days after exposure. Each plot contains the first two letters of the subject identifier, sex, and age at exposure in d.o. with black symbols representing middle-aged mice and red symbols representing old-aged mice. The dashed vertical line at day 0 represents noise exposure day. The dashed horizontal line in ***C*** and ***D*** represents pre-exposure thresholds at 0 dB for each mouse.

**Figure 2.**
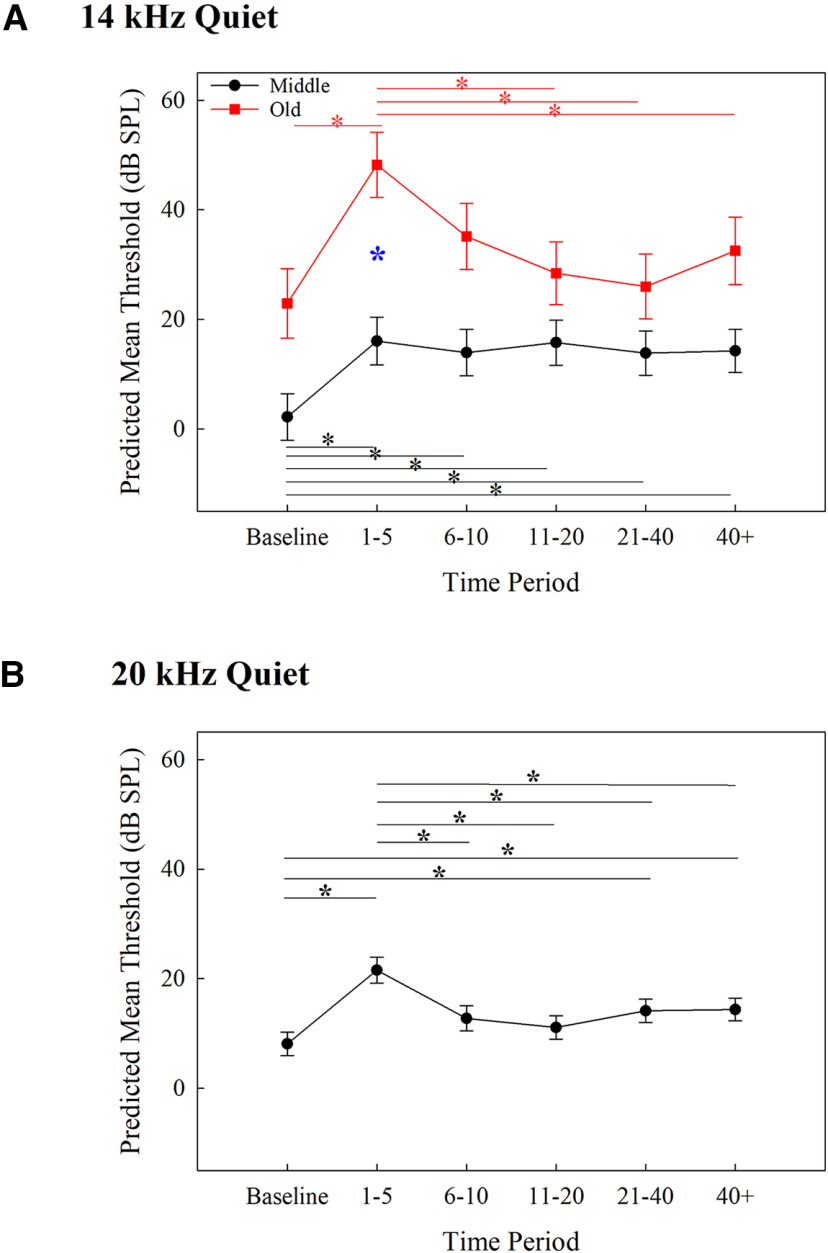
Predicted mean thresholds for tone detection in quiet from the mixed-effects model for 14 (***A***) and 20 (***B***) kHz across each time period (corrected *p *= 0.0014). Middle-aged mice are represented by black circles and within-group significant comparisons are designated with black lines and *. Old-aged mice are represented by red squares and within-group significant comparisons are designated with red lines and *. The blue * in ***A*** represents significant between age groups comparisons. Error bars represent SEM.

Individual absolute thresholds (dB SPL) and threshold shifts (dB) for middle-aged and old-aged mice over time are shown in Figure 1 (see Table 1 for statistical table). The statistical model for the detection of 14 kHz in quiet revealed a significant main effect of age group (*F *=* *10.42, *p *=* *0.0098), time period (*F *=* *16.61, *p *=* *3.16 × 10^−15^), and interaction between age group and time period (*F *=* *7.70, *p *=* *5.39 × 10^−7^ (for results, see Fig 2*A*)). *Post hoc* comparisons across age groups reveal old-aged mice had significantly higher thresholds than middle-aged mice 1–5 d after noise exposure (*p *=* *0.0004). Within age groups, middle-aged mice had significantly lower thresholds at baseline than at all other time points (*p *<* *0.0001). Old-aged mice had significantly lower thresholds at baseline than at 1–5 d (*p *<* *0.0001), and significantly higher thresholds at 1–5 d than at 11–20 d (*p *<* *0.0001), 21–40 d (*p *<* *0.0001), and over 40 d (*p *=* *0.0002). These results reveal that for 14-kHz tones in quiet, detection thresholds for older mice were more affected by noise exposure than middle-aged mice (significant difference only at 1–5 d across age groups and a larger increase in threshold from baseline in old-aged mice than middle-aged mice), but old-aged mice recovered rapidly from that increase while middle-aged mice did not.

**Table 1:** Statistical table for all behavioral modeling including *F* values, degrees of freedom (numerator, denominator), *p* values, and effect sizes (*η_p_^2^*) for quiet (left) and masked (right) conditions separately for 14 kHz (top) and 20 kHz (bottom)

	Quiet				Masked			
	*F*	df	*p*	*η_p_^2^*	*F*	df	*p*	*η_p_^2^*
14 kHz								
Age group	10.42	1, 9.40	**0.0098**	0.079004	1.36	1, 6.06	**0.287**	0.0106
Time period	16.61	5, 517.76	**3.16e-15**	0.629355	21.85	5, 395.85	**< 2.2e-16**	0.8506
Age group:time period	7.70	5, 517.76	**5.39e-07**	0.291641	3.56	5, 395.85	**0.004**	0.1387
20 kHz								
Age group					0.0492	1, 6.01	0.8319	0.0005
Time period	11.75	5, 234.48	**3.80e-10**		10.01	5, 373.08	**5.26e-9**	0.5059
Age group:time period					9.77	5, 373.08	**8.69e-09**	0.4936

Significant values are bolded.

#### 20 kHz in quiet

**Figure 3.**
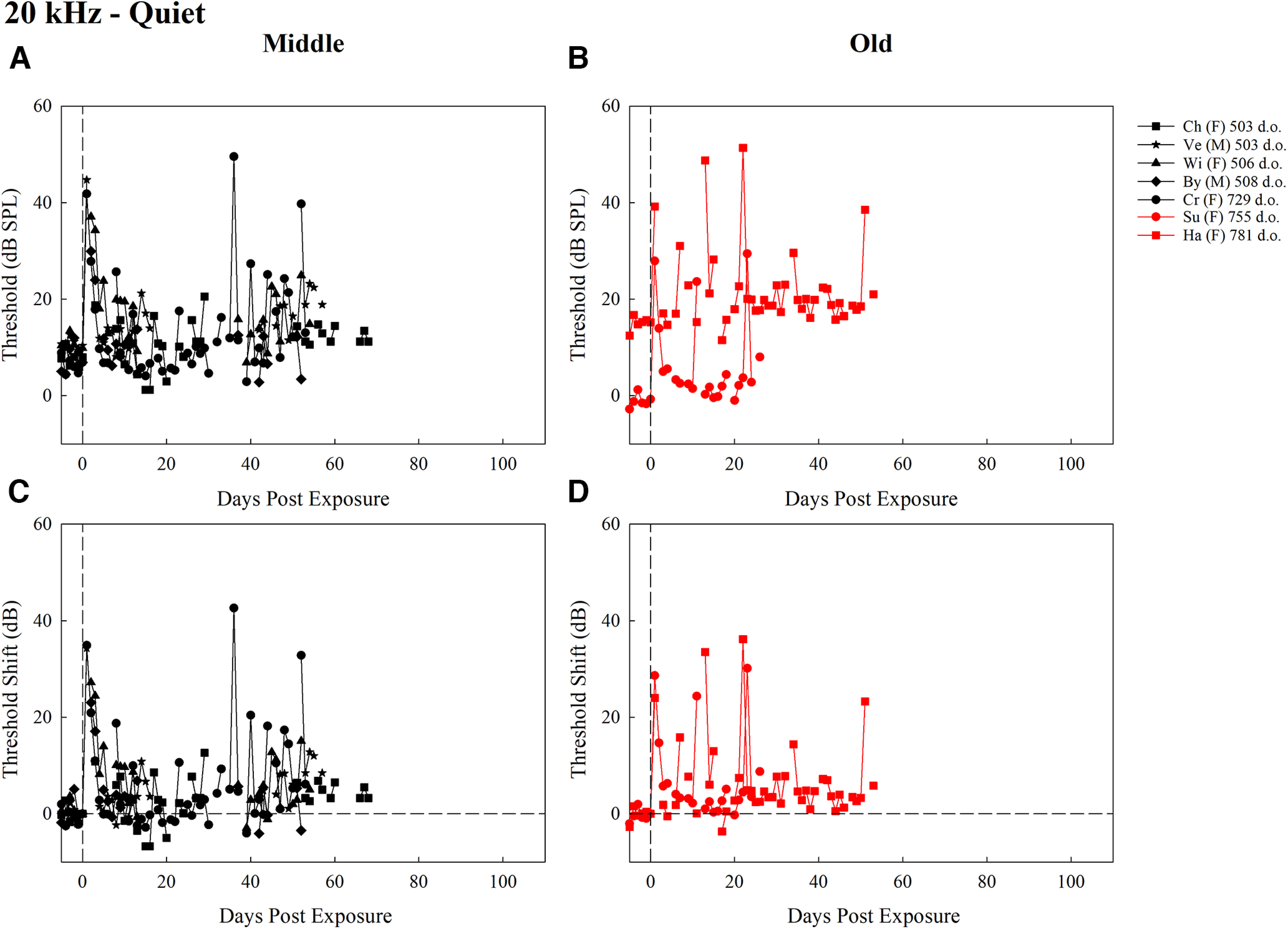
Thresholds (dB SPL) for middle-aged mice (***A***) and old-aged mice (***B***), and threshold shifts (dB) for middle-aged mice (***C***) and old-aged mice (***D***), trained to detect 20-kHz tones in quiet for all test days after exposure. Each plot contains the first two letters of the subject identifier, sex, and age at exposure in d.o. with black symbols representing middle-aged mice and red symbols representing old-aged mice. The dashed vertical line at day 0 represents noise exposure day. The dashed horizontal line in ***C*** and ***D*** represents pre-exposure thresholds at 0 dB for each mouse.

Individual absolute thresholds (dB SPL) and threshold shifts (dB) for middle-aged and old-aged mice over time are shown in Figure 3. The statistical model for the detection of 20-kHz tones in quiet revealed a significant main effect of time period (*F *=* *11.75, *p *=* *3.80 × 10^−10^) since the model with the best fit only included time period as a factor (for results, see Fig. 2*B*). *Post hoc* comparisons revealed that thresholds at baseline were significantly lower than at 1–5 d (*p *<* *0.0001), 21–40 d (*p *=* *0.002), and over 40 d (*p *=* *0.0005). Thresholds were significantly higher at days 1–5 than at 6–10 (*p *=* *0.0004), 11–20 (*p *<* *0.0001), 21–40 (*p *=* *0.0013), and over 40 d (*p *=* *0.0012). No other comparisons were significantly different. These findings reveal that the noise exposure led to a temporary increase in thresholds which improved marginally but did not return to baseline sensitivity, regardless of the age of the subject.

### Behavior, masked

#### 14 kHz in noise

**Figure 4.**
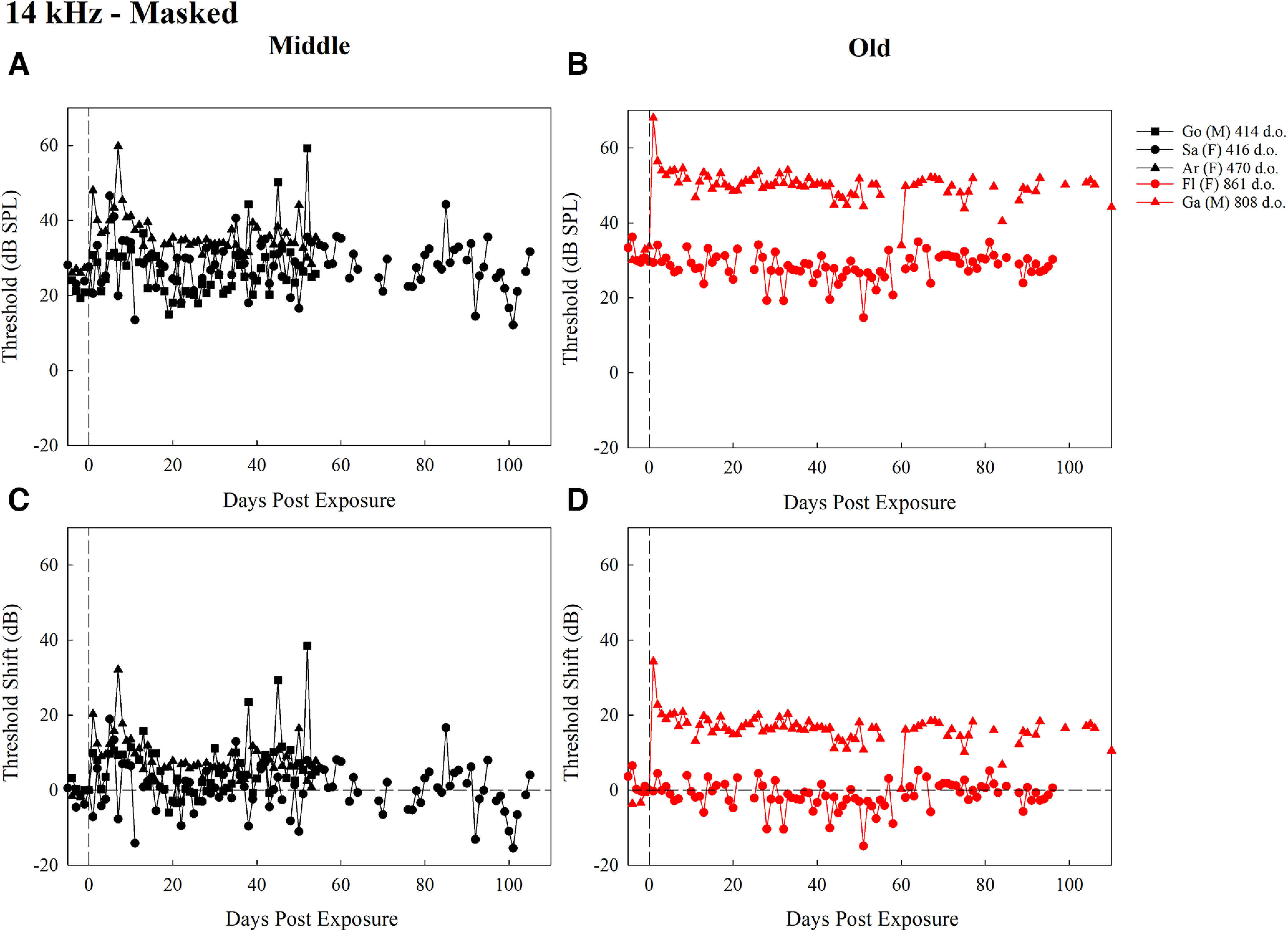
Thresholds (dB SPL) for middle-aged mice (***A***) and old-aged mice (***B***), and threshold shifts (dB) for middle-aged mice (***C***) and old-aged mice (***D***), trained to detect 14-kHz tones in a masker for all test days after exposure. Each plot contains the first two letters of the subject identifier, sex, and age at exposure in d.o. with black symbols representing middle-aged mice and red symbols representing old-aged mice. The dashed vertical line at day 0 represents noise exposure day. The dashed horizontal line in ***C*** and ***D*** represents pre-exposure thresholds at 0 dB for each mouse.

**Figure 5.**
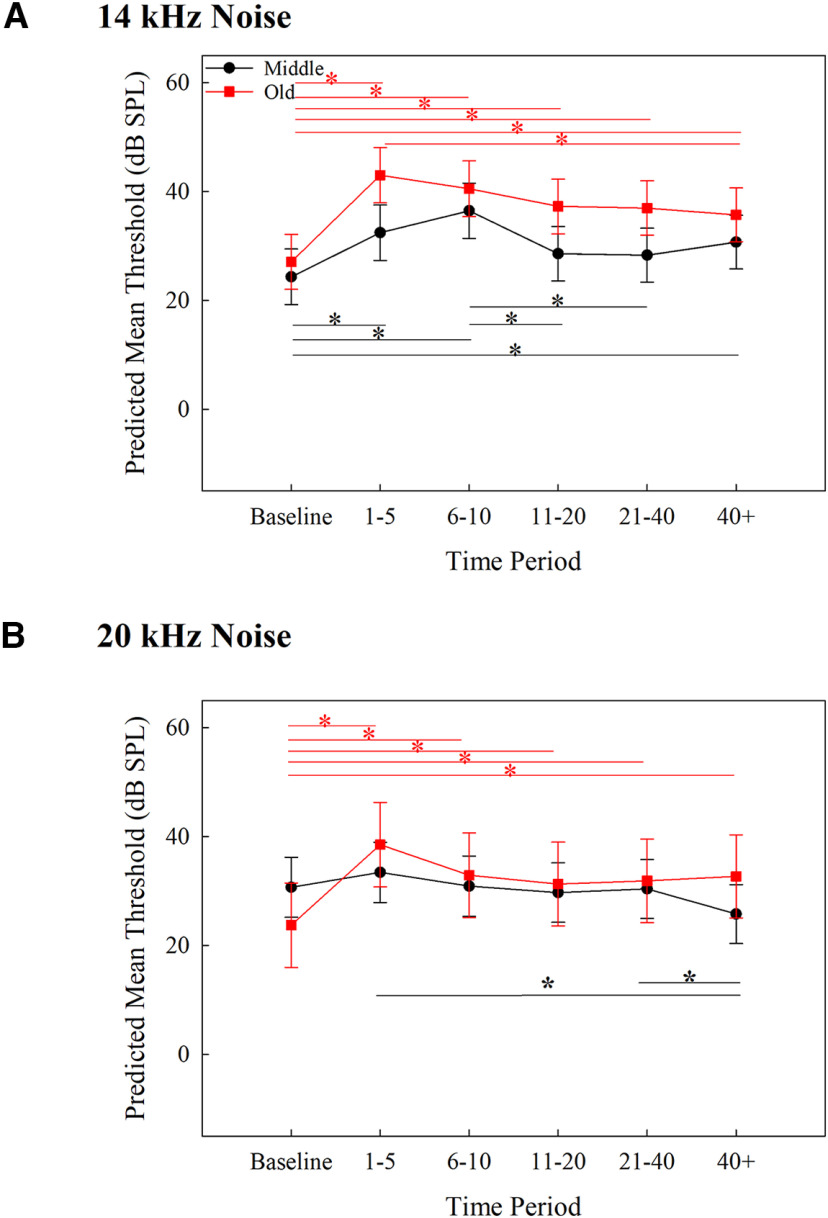
Predicted mean thresholds for tone detection in noise from the mixed-effects model for 14 (***A***) and 20 (***B***) kHz across each time period (adjusted *p *= 0.0014). Middle-aged mice are represented by black circles and within-group significant comparisons are designated with black lines and *. Old-aged mice are represented by red squares and within-group significant comparisons are designated with red lines and *. Error bars represent SEM.

The masked thresholds (dB SPL) and threshold shifts (dB) for middle-aged and old-aged mice over time are represented in Figure 4. The statistical model for the detection of a 14-kHz tone in a noise masker revealed a significant main effect of time period (*F *=* *21.85, *p *<* *2.2 × 10^−16^) and interaction between age group and time period (*F *=* *3.56, *p *=* *0.004; Fig. 5*A*). Across the age groups, there were no significant differences within each time period (*p *>* *0.0014). In middle-aged mice, baseline thresholds were significantly lower than at 1–5 d (*p *= 0.001), 6–10 d (*p *<* *0.001), and over 40 d (*p *=* *0.001). Thresholds were significantly higher at 6–10 d than at 11–20 d (*p *=* *0.0001), and 21–40 d (*p *<* *0.0001). For old-aged mice, baseline thresholds were significantly lower than all other time periods (*p *<* *0.0001), and thresholds were significantly higher at 1–5 d than over 40 d (*p *=* *0.0001). No other contrasts were significantly different. These differences reflect that while thresholds did not return to baseline (evidenced by significant differences between baseline and all other time periods in the old-aged group and baseline and several intermediate and late time points in the middle-aged group), there was some recovery from the initial threshold shifts in both groups (evidenced by the significant decrease in thresholds between 6–10 and over 40 d in the old-aged group and the lack of a significant difference between baseline and 11–20 and 21–40 d in the middle-aged group).

#### 20 kHz in noise

**Figure 6.**
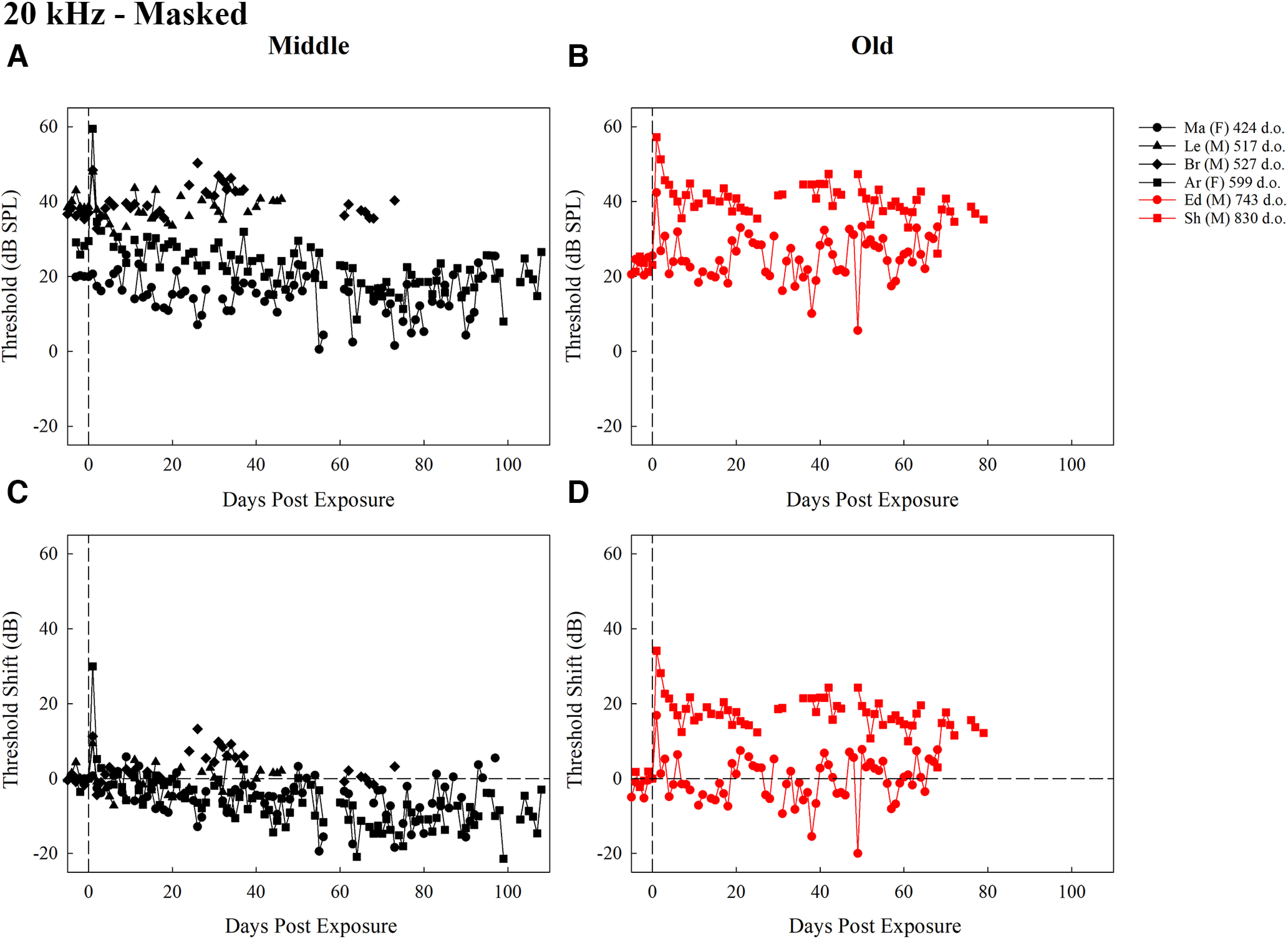
Thresholds (dB SPL) for middle-aged mice (***A***) and old-aged mice (***B***), and threshold shifts (dB) for middle-aged mice (***C***) and old-aged mice (***D***), trained to detect 20-kHz tones in a masker for all test days after exposure. Each plot contains the first two letters of the subject identifier, sex, and age at exposure in d.o. with black symbols representing middle-aged mice and red symbols representing old-aged mice. The dashed vertical line at day 0 represents noise exposure day. The dashed horizontal line in ***C*** and ***D*** represents pre-exposure thresholds at 0 dB for each mouse.

The masked thresholds (dB SPL) and threshold shifts (dB) for middle-aged and old-aged mice over time are represented in Figure 6. The statistical model for the detection of 20 kHz in a masker revealed a significant main effect of time period (*F *=* *10.01, *p *=* *5.26 × 10^−9^), and a significant interaction between age group and time period (*F *=* *9.77, *p *=* *8.69 × 10^−9^; for model findings, see Fig. 5*B*). *Post hoc* comparisons were conducted comparing middle-aged and old-aged mice at each of the time periods. No significant differences emerged when comparing across the age groups for any time period (*p *>* *0.0014). Within middle-aged mice, thresholds from 1 to 5 d were significantly higher than those over 40 d (*p *<* *0.0001), and thresholds were significantly higher at 21–40 d than over 40 d (*p *<* *0.0001). This result is striking because middle-aged mice were generally improving in thresholds over time while not experiencing much change in sensitivity after noise exposure. In contrast, old-aged mice had significantly lower thresholds at baseline than at all other time periods (*p < *0.001) suggesting the noise exposure had a greater effect in this group. No other comparisons were significant. These results suggest that for detecting 20-kHz tones in a noise masker, old- but not middle-aged mice had mildly disrupted detection after noise exposure. The lack of a significant main effect of age group here suggests that although our old group was affected by the noise exposure and our middle-aged group was not, the effects of the noise exposure were minor in the old subjects for the detection of 20 kHz in a masker.

### Behavior, threshold variability

**Figure 7.**
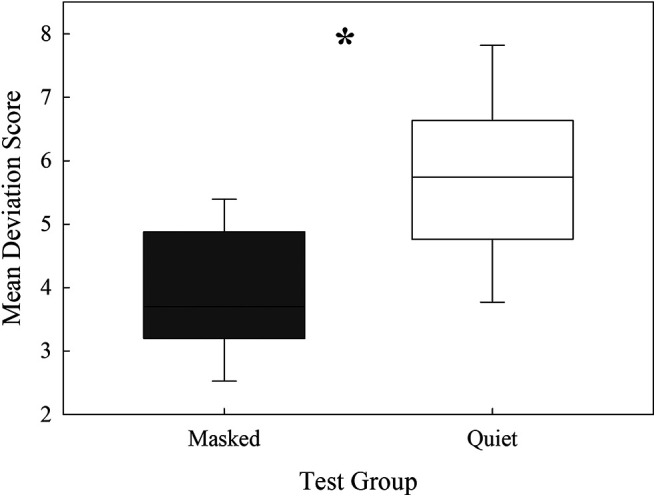
Mean deviation scores after exposure for masked and quiet listening conditions (*p < 0.05). Box height represents the 75th percentile (Q3), the horizontal line within box represents the median, the bottom of the box represents the 25th percentile (Q1) and the error bars represent represent the maximum observation that falls within the upper limit (Q3+1.5(Q3−Q1)) and the minimum observation that falls within the lower limit (Q1−1.5 (Q3−Q1)).

An ANOVA comparing the interaction between masker status (quiet or noise) and frequency (14 or 20 kHz) revealed a significant main effect of masker status (*F *= 15.35, *p *=* *0.00,074). A *post hoc* Tukey test revealed that there was significantly more variability when mice were detecting signals in quiet than in noise (*p *=* *0.0007; Fig. 7; Table 2). Linear regression analyses examining the relationship between these factors and the age of the mouse did not reveal significant results, reflecting similar effects of noise exposure on threshold stability across the age ranges tested (see Table 2). The significant results of the threshold variability analyses indicate the background noise masker had a stabilizing effect on sound detection in noise-exposed mice.

**Table 2:** Statistical table for all day-to-day variability measurements, including an ANOVA analysis examining the interaction between masker status and stimulus, a *post hoc* Tukey’s test, and linear regressions examining whether the age of the mouse (in d.o.) could predict changes in day-to-day variability

Test type	Comparisons/independent variables	Test statistic	SS	df	*p* value	Effect size
ANOVA	After <- stimulus × masker status	*F* value			* *	*η_p_^2^*
	Stimulus	0.843	1.176	1	0.3685	0.0220009
	Masker status	15.353	21.42	1	**0.00074**	0.400876
	Stimulus × masker status	0.103	0.143	1	0.7515	0.002676
	Residuals		30.694	22		
		Difference score				Cohen’s *d*
Tukey’s HSD	Quiet vs masked after noise exposure	1.821			**0.00074**	1.591433
		*F* value				*R* ^2^
Linear regression	Quiet before noise exposure	1.07		(2, 12)	0.3213	0.0819
	Noise before noise exposure	1.3096		(2, 10)	0.2791	0.1158
	Quiet after noise exposure	2.5804		(2, 12)	0.1342	0.177
	Noise after noise exposure	1.2205		(2, 10)	0.2951	0.1088
	14 kHz in quiet after	0.2825		(2, 5)	0.6178	0.0535
	14 kHz in noise after	1.9356		(2, 4)	0.2365	0.3261
	20 kHz in quiet after	5.076		(2, 5)	0.074	0.5038
	20 kHz in noise after	0.2996		(2, 4)	0.6132	0.0697

Significant values are bolded throughout and relevant effect sizes are presented.

### Cochlear immunohistochemistry

We compared cochlear degeneration patterns to determine whether noise exposure further damaged the cochlea beyond what occurs with normal aging. Data from aged, unexposed mice contained a merged dataset of new subjects (*N* = 3) and our previously published data (Kobrina et al., 2020) in aged, unexposed subjects processed under similar conditions (*N* = 7) to increase power. All data from unexposed mice analyzed in the current experiment (*N* = 10) were from subjects tested under the same conditions, and all tissues were processed by the same experimenter using the same procedures and reagents.

**Figure 8.**
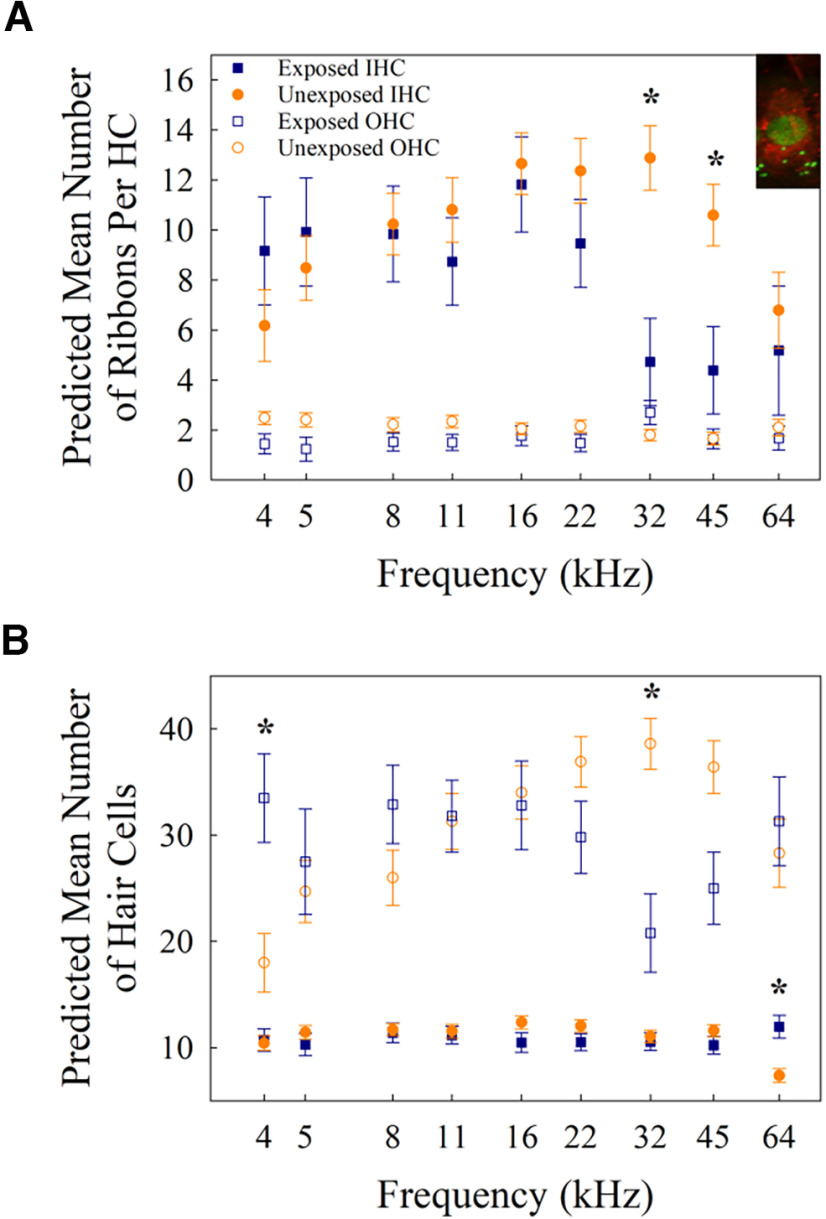
***A***, Predicted mean number of ribbons per hair HC across frequencies for unexposed (orange) and exposed (blue) mice. Error bars are standard error and *p < 0.0056. The top right corner includes sample cochlear immunohistochemistry with Myosin 6 (red) to visualize the hair cell and CTBP2 (green) to visualize ribbons. ***B***, Predicted mean number of hair cells per 100 μm at each frequency for IHCs (closed symbols) and OHCs (open symbols) in unexposed (orange) and exposed mice (blue). Error bars are standard error and *p < 0.0056.

On the full dataset (*N* = 15) of unexposed (*N* = 10) and exposed mice (*N* = 5), models were constructed to evaluate each dependent variable (number of IHCs per 100 μm, number of OHCs per 100 μm, number of ribbons per IHC, and number of ribbons per OHC) separately, as they are predicted by the frequency (4, 5, 8, 11, 16, 22, 32, 45, and 64 kHz) and noise-exposure status (exposed or unexposed), and taking into account the individual mouse ID and comparing to intercept-only models for significance and goodness of fit. For sample cochlear immunostained tissue, see the Figure 8*A*, top right corner. Age was included in the test models but the mean age in the exposed and unexposed groups did not differ (unexposed M = 775.5, range: 639–889 d.o., exposed M = 615, range: 424–844 d.o.). Therefore, models including age as a factor did not have the highest goodness of fit for the statistical model.

### Number of ribbons per hair cell

The linear mixed-effects model comparing the number of ribbons per IHC revealed a significant main effect of frequency (*F *=* *3.81, *p *=* *0.0006) and a significant interaction between frequency and exposure group (*F *=* *3.67, *p *=* *0.0009; for summary of anatomic statistical tests, see Table 3). *Post hoc* analyses revealed that noise-exposed mice had significantly fewer ribbons per IHC than unexposed mice at 32 (*t* = −3.77, *p *=* *0.0003) and 45 kHz (*t* = −2.8 90, *p *=* *0.0048; see Fig. 8*A*). No other comparisons at specific frequency regions differed significantly between exposed and unexposed mice (*p *>* *0.0056). The model comparing number of ribbons per OHC revealed a significant interaction between frequency and exposure group (*F *=* *2.31, *p *=* *0.028); however, none of the *post hoc* analyses were statistically significant (*p *>* *0.0056). Given the small number and limited range of ribbons per OHC (approximately one to two), the biological significance of the interaction may be negligible.

**Table 3:** Statistical table for all anatomic tests including *F* values, degrees of freedom (numerator, denominator), *p* values, and effect sizes (*η_p_^2^*) for number of IHCs (top), number of OHCs (second down), number of puncta per IHC (third down), and number of puncta per OHC (bottom)

Test	Variables	*F*	df	*p*	*η_p_^2^*
	Frequency	1.1512	8, 116	0.334901	0.264604
Number of IHCs	Exposure group	0.4954	1, 116	0.482941	0.014234
	Frequency:Exposure group	3.1375	8, 116	**0.003005**	0.721163
	Frequency	1.9385	8, 92.652	0.0633	0.200283
Number of OHCs	Exposure group	0.1575	1, 15.553	0.6969	0.002036
	Frequency:Exposure group	7.7205	8, 92.652	**7.00E-08**	0.797681
	Frequency	3.8095	8, 101.521	**0.000599**	0.489336
Number of puncta per IHC	Exposure group	2.4514	1, 15.805	0.137217	0.039362
	Frequency:Exposure group	3.6692	8, 101.521	**0.000854**	0.471303
	Frequency	0.6406	8, 81.208	0.74136	0.187705
Number of puncta per OHC	Exposure group	3.6937	1, 14.988	0.07384	0.135288
	Frequency:Exposure group	2.3105	8, 81.208	**0.02759**	0.677006

Significant values are bolded.

### Total number of hair cells per 100 μm

The linear mixed-effects model comparing the total number of IHCs per 100 μm revealed a statistically significant interaction between frequency and exposure group (*F *=* *3.14, *p *=* *0.003). *Post hoc* analyses revealed that mice in the noise-exposed group had significantly more IHCs at 64 kHz than unexposed mice (*t *=* *3.71, *p *=* *0.0003; see Fig. 8*B*). No other comparisons at specific frequencies between exposed and unexposed mice differed significantly (*p *>* *0.0056). The statistical model comparing the total number of OHCs per 100 μm revealed a statistically significant interaction between frequency and exposure group (*F *=* *7.72, *p *=* *7.0 × 10^−8^). *Post hoc* analyses revealed that noise-exposed mice had significantly more OHCs at 4 kHz than unexposed mice (*t *=* *3.09, *p *=* *0.0026) and significantly more OHCs at 32 kHz in unexposed than noise exposed mice (*t* = −4.03, *p *=* *0.0001). No other comparisons at specific frequencies between exposed and unexposed mice differed significantly (*p *>* *0.0056).

## Discussion

Our data show that noise exposures used to induce auditory nerve synaptopathy in young adult mice lead to permanent changes in behavioral hearing sensitivity for pure tones in old-aged mice, and milder changes in middle-aged mice when listening in quiet and noise. Although the temporary hearing loss was overt and apparent in both quiet and masked conditions, behavioral hearing sensitivity was highly variable between mice and recovered to varying degrees over time. Unexpectedly, detection performance was more stable across time when listening in the presence of a noise masker compared with quiet listening conditions. Overall, greater high-frequency IHC ribbon loss and OHC loss was observed in exposed mice compared with degeneration associated with age alone, but this damage occurred in frequency regions that were outside the regions corresponding to the tone stimuli used in the behavioral detection experiments.

### Hearing sensitivity in quiet

The behavioral experiments revealed interesting interactions between age group and time period after noise exposure. Similar noise exposure parameters have previously been shown to induce temporary auditory brainstem response threshold shifts and permanent loss of auditory nerve synapses in young adult mice without permanent threshold shifts (for review, see Kujawa and Liberman, 2015). We observed increased behavioral thresholds after noise exposure that generally returned to baseline or near-baseline sensitivity over time in middle-aged mice. In contrast, performance typically did not fully return to baseline in old-aged mice even if sensitivity improved relative to the initial shift. Similar effects have been shown previously in aged rats that sustained permanent auditory brainstem response thresholds shifts after noise exposure that only resulted in temporary threshold shifts in younger rats (Wang et al., 2011). These results suggest that age hinders recovery of hearing sensitivity after damaging noise exposure.

### Hearing sensitivity in noise

For detection of tones in noise, there were significant differences across age and time period after exposure. Detection of 14-kHz tones in noise temporarily worsened after noise exposure in most of the mice tested, whereas performance worsened in old-aged but not middle-aged mice tested on the 20 kHz in noise condition. A previous behavioral study in NMRI mice showed that noise maskers were less effective in aged animals compared with young ones, and a similar effect may be at play in the present study (Ehret, 1979). Cortical reorganization may have occurred in these mice, although it begs the question of why it did not happen in other testing conditions or to the other mice tested in this condition. Regardless, 14-kHz tone detection was severely affected by noise exposure across the lifespan, whereas middle-aged mice experienced less severe deficits than old-aged mice for 20 kHz.

### Performance stability

Mice showed high variability in day-to-day hearing sensitivity after noise exposure when tested in quiet. Animals’ performance was more stable in the presence of a noise masker than in the quiet condition, which was an unexpected result (Fig. 7). We are unaware of any other noise exposure studies examining the relationship between threshold stability after noise exposure, so we can only speculate about why this effect was observed. One possible explanation for our finding is that increased hearing stability emerged because of stochastic resonance, where the presence of a fluctuating noise increases detection performance by stabilizing internal noise (Chatterjee and Robert, 2001; McDonnell and Abbott, 2009). The so-called “noise benefit” observed in our behavioral detection data may also occur as central processes that are otherwise disengaged in quiet become engaged in the presence of a noise masker. This could reflect activation of medial olivocochlear pathways that enhance the neural representation of tones in the presence of background noise, although the perceptual hearing in noise benefits of olivocochlear activation remain uncertain (Lauer et al., 2021). Noise exposure induces plasticity in dopaminergic lateral olivocochlear neurons, and this may have a stabilizing effect on the representation of tones in noise in the auditory nerve (JS Wu et al., 2020). The noise background could also have activated medial olivocochlear neurons which may become hyperactive in the damaged auditory system (Knudson et al., 2014).

We did not observe the instability of detection in noise that reportedly occurs before overt threshold shifts in C57BL/6 mice with hereditary, early-onset hearing loss (Prosen et al., 2003), possibly because of the abrupt nature of noise-induced hearing damage. Intriguingly, the C56BL/6 strain shows weak medial olivocochlear activity compared with CBA mice even at young ages, although both strains exhibit medial olivocochlear degeneration with age (Frisina et al., 2007; Sinclair et al., 2017; Vicencio-Jimenez et al., 2021).

In humans, cognitive processes such as attention are critical for detecting a stimulus in masked environments (Strait and Kraus, 2011), thus the decreased day-to-day variability of detection performance in noise might reflect activation of attentional pathways by noise. This phenomenon has been observed in other species. High levels of anthropogenic masking noises that create difficult listening conditions lead to increased vigilance in birds (Evans et al., 2018). It is important to note that we avoided the use of louder background noise levels because, in our experience, mice are less inclined to engage in the operant task when ambient noise levels are too high. Relatedly, background noise can facilitate the acoustic startle response in mice (Ison, 2001; McGuire et al., 2015; Kim et al., 2022), presumably reflecting increased vigilance or fear. The role of attention and vigilance in hearing in noise should be investigated further in future studies.

### Cochlear damage

Our anatomical analysis showed that noise-exposed mice experienced loss of OHCs and a reduction in the number of ribbons per hair cell in the high-frequency regions of the cochlea relative to what occurs with aging alone. OHCs in the base of the cochlea are most vulnerable to the effects of both noise and age, and impaired recovery from noise exposure in the aging auditory periphery has been previously reported (Möhrle et al., 2016). Thus, the confluence of noise and age resulting in more anatomical and perceptual damage in the present study is not surprising.

Increased vulnerability of the aged auditory system may be due to a reduced capacity for recovery or an increased susceptibility to damage in weakening cells (Ohlemiller et al., 2000). Although age and noise exposure have previously been shown to have additive effects (Sun et al., 1994), age was not statistically associated with cochlear damage in our study, likely because of the similarity in the ages of our exposed and unexposed groups and the range of ages (400–700 d.o.) of mice tested. Remarkably, the regions with increased damage were outside the mid-frequency regions of the tones used in the behavioral detection task. However, a change in the cochlear place-frequency map may have occurred in aged, noise-exposed mice. Noise exposure is known to shift the characteristic frequencies of auditory nerve fibers down by ∼0.6–1.2 octaves in young adult mice (Müller and Smolders, 2005). Whether or not similar shifts occur in aged, noise-exposed mice or aged mice alone is uncertain. Nevertheless, a down-shift in auditory nerve fiber tuning would presumably not have had a negative impact on the detection of tones in the mid-frequency range since the number of ribbons and hair cells was not affected by noise for regions that are 0.6–1.6 octaves below the tone frequencies.

One surprising finding regarding the cochlear damage patterns was the increased OHC survival in aged, noise-exposed mice in the apical-most region of the cochlea and the increased survival in aged, noise-exposed mice in the basal-most region of the cochlea relative to unexposed, aged controls. One possible explanation for these findings could be that experience with nondamaging sounds can promote cell survival with age (Dziorny et al., 2021). Thus, daily exposure to sounds in the test environment may have protected against apical OHC and basal IHC loss. Sound exposure may activate olivocochlear efferents, and the medial olivocochlear neurons, in particular, have protective effects in the lower frequency regions of the aging cochlea (Liberman et al., 2014). We can consider how experience with noise may alter the degree of damage sustained by the aging auditory system, but further research is necessary to understand the underlying mechanisms.

In conclusion, our experiments revealed that behavioral recovery from damaging noise exposure is dependent on age and listening conditions. Aged noise-exposed mice were more susceptible to hearing loss when detecting a tone in quiet than when detecting a tone embedded in a masker. Finally, post-exposure mice listening to 14-kHz tones experienced greater threshold shifts than mice listening to 20-kHz tones. Together, these results highlight the importance of employing behavioral methodologies and a longitudinal design in examining the complex interactions between noise-induced and age-related hearing losses, as well as assessing hearing in both quiet and noise.

## Synthesis

Reviewing Editor: Christine Portfors, Washington State University Decisions are customarily a result of the Reviewing Editor and the peer reviewers coming together and discussing their recommendations until a consensus is reached. When revisions are invited, a fact-based synthesis statement explaining their decision and outlining what is needed to prepare a revision will be listed below. The following reviewer(s) agreed to reveal their identity: Christine Köppl, Kenneth Henry.

The authors' responsive revision of their manuscript on behavioral tone detection in aging noise-exposed mice is greatly appreciated. The reviewers find the revised manuscript more clear and easier to follow. The presentation of the results however still has problems that need to be addressed. In particular, there is concern over the model generating data at mouse ages that were not directly evaluated and statistical concerns/errors that may affect the interpretation of the results.

Detailed comments:

The title is confusing to me; how can ‘behavioral tone detection’ have ‘temporary and permanent effects’ on something? Perhaps ‘Effects of noise exposure and aging on mouse behavioral tone detection in quiet and in noise’?

Line 234 Clarify that age^2 was a continuous independent variable.

Line 240. It seems like thresholds were “predicted” at different ages from the linear model, which can be dangerous when there is little or no data at the age for which the prediction is made (see more on this below). Some additional description of the procedure would be helpful here if it is retained, since post-hoc comparisons are more typically made between groups defined based categorical independent variables. How do results/inferences differ with a categorical variable?

Line 296 and 298. Here and elsewhere the P values are incorrect and do not match those presented in the tables at the end of the paper. The exponents appear to have dropped off. It’s a little unusual to see exponents on the P values. Oftentimes for p value below some very small criterion value (e.g, 0.0001), P values are simply reported as ‘P < 0.0001’.

Line 294-307. It is hard to see the support for the statement on line 304-307 that young mice were less affect by the noise exposure, with greater recovery. The model results in 2A show more of a main effect of age, but with more variability in the young ‘group’ resulting in no significant differences across time periods. But this is not actually a group (the text in this section needs to make this much clearer), it is the predicted pattern for 500 day-old mice. I don’t understand why the model would predict greater variability? Does this have to do with the fact that no mice in this experiment are that young, and only 2 are between 500 and 600 days at the age of exposure? It would be comforting to go back to the raw data and see what patterns the individual mice are showing, but this not possible due to the density of Fig 1B. One alternative would be to show data from 450-550, 551-650, and 651-750 day-old mice in separate panels. Perhaps 1A could be removed and the only lost information (mean baseline thresholds) inserted into the legend of the new panels.

Figure 2. ‘Predicted mean threshold’ might be a clearer title for the y axis here and elsewhere.

Line 322-346. First, there is another incorrect p value on line 325 that will be disconcerting to readers. Second, the same issue exists of having a detailed comparison of hearing loss at different age ranges based on a statistical model prediction, except here, it is much worse because 3 mice are 414-470 days old and two mice are old (>800 days). One would have to have extreme faith in age^2 to draw any conclusion about what is happening in 600 day-old mice, but a mean line appears in Fig 5A along with error bars and detailed discussion of the pattern in the text. An even larger issue is that the statistical model results (predicted thresholds) in 5A are totally different than the raw data in Fig. 4. The threshold shift immediately after noise is ∼10 dB in the raw data but the model predicted threshold is 20-40 dB

Line 348-364. It is a little surprising that so many pairwise comparisons are significant given the overlapping standard error bars in Fig. 5B, once again casting doubt on the validity of inferences from the model.

Line 367 Some introduction to this section would be helpful. Also, authors often report the p values for the non-significant main effects to establish why they were dropped from the statistical model

Please clarify the chemicals used:

Line 171: anaesthetic doses are given in mg/kg body weight. This is perfectly clear. But what is meant by “ ... at 100% of their body weight”?

line198-199: PBS (phosphate-buffered saline) is usually the carrier solution in which all other substances are contained at the concentrations given. As it reads now, the PBS itself is diluted to 10% of its original strength. Is this correct?
